# Association of high-sensitivity troponin with advanced fibrosis in metabolic dysfunction-associated steatotic liver disease and the potential mediating role of inflammation: A cross-sectional study of NHANES, 1999 to 2004

**DOI:** 10.1097/MD.0000000000049537

**Published:** 2026-07-03

**Authors:** Chun Yu Li, Yun Xiu Wu, Qing Zhang, Kai Wang

**Affiliations:** a Department of Gastroenterology, Guangdong Provincial People’s Hospital, Zhuhai Hospital (Jinwan Central Hospital of Zhuhai), Zhuhai, Guangdong, China; b Department of Endocrinology, Guangdong Provincial People’s Hospital, Zhuhai Hospital (Jinwan Central Hospital of Zhuhai), Zhuhai, Guangdong, China; c Department of Geriatrics, The Affiliated Taizhou People’s Hospital of Nanjing Medical University, Taizhou, Jiangsu, China; d Department of Gastroenterology, The Affiliated Taizhou People’s Hospital of Nanjing Medical University, Taizhou, Jiangsu, China.

**Keywords:** advanced liver fibrosis, hs-troponin T, inflammation marker, metabolic dysfunction-associated steatotic liver disease, NHANES

## Abstract

Given the high prevalence of metabolic dysfunction-associated steatotic liver disease (MASLD) and its severe adverse hepatic outcomes, risk stratification for patients with MASLD is crucial. Notably, cardiovascular disease (CVD) represents the leading cause of mortality in this population. High-sensitivity troponin (hs-troponin) is a well-established biomarker of subclinical myocardial injury and predicts cardiovascular outcomes. However, its specific relationship with advanced liver fibrosis (ALF) in MASLD remains unexplored. Therefore, we investigated this association in individuals with MASLD without CVD. We analyzed data from 4684 adults aged ≥18 years in the 1999 to 2004 National Health and Nutrition Examination Survey. Multivariable logistic regression examined associations between hs-troponin and ALF. Diagnostic utility was assessed using receiver operating characteristic curve analysis. Moreover, we conducted a mediation analysis to explore the role of inflammation, as represented by the monocyte-to-lymphocyte ratio, in the aforementioned association. Among 4684 patients without CVD, 169 had ALF. After controlling for confounding factors, hs-troponin T showed a significant positive association with ALF (adjusted odds ratio: 1.02, 95% confidence interval: 1.01–1.03, *P* = .020). Additionally, receiver operating characteristic analysis indicated that at a cutoff of 7.28 ng/L, hs-troponin T predicted ALF with an area under the curve of 0.85 (95% confidence interval: 0.82–0.88). Further mediation analysis revealed that inflammation partially mediated the association between hs-troponin T and ALF. Our findings indicate that elevated hs-troponin T is independently associated with a higher risk of ALF, which can assist clinicians in risk stratification for this population. These findings suggest that hs-troponin T, a marker of subclinical cardiovascular stress, may also serve as a practical tool for hepatic risk stratification.

## 1. Introduction

Metabolic dysfunction-associated steatotic liver disease (MASLD) has emerged as the most prevalent chronic liver disease, affecting approximately 38% of adults worldwide.^[1]^ MASLD is considered a hepatic manifestation of metabolic syndrome, is closely related to the systemic immune-inflammatory state, and disease progression is significantly heterogeneous.^[2]^ Notably, cardiovascular disease (CVD) represents the leading cause of mortality in patients with MASLD, accounting for nearly one-third of all deaths.^[3]^ Several studies have confirmed an epidemiological association between MASLD and an increased risk of CVD, an association that is independent of traditional cardiovascular risk factors.^[4,5]^ Therefore, identifying reliable biomarkers is crucial for recognizing patients with MASLD who are at high risk of poor outcomes, enabling early risk stratification and targeted interventions to improve prognosis.

High-sensitivity troponin (hs-troponin) testing has demonstrated strong capability in detecting subclinical myocardial injury and predicting adverse cardiovascular outcomes.^[6]^ Previous studies have highlighted the prognostic value of hs-troponin across various populations, confirming its potential as a tool for cardiovascular risk stratification.^[7–9]^ Furthermore, recent research has observed an association between hs-troponin levels and both all-cause and cardiovascular mortality in patients with MASLD, irrespective of concurrent CVD.^[10,11]^ Notably, prior studies have also shown that hs-troponin is related to the severity and prognosis of patients with alcoholic cirrhosis^[12]^ and that troponin levels may be mildly elevated in cirrhotic cardiomyopathy, indicating ongoing myocardial injury.^[13]^ The link between MASLD and CVD is believed to involve intricate pathophysiological mechanisms, including the release of hepatic inflammatory cytokines, lipotoxicity, and insulin resistance.^[14,15]^ Once advanced liver fibrosis (ALF) develops, it may further elevate CVD risk by exacerbating systemic metabolic dysregulation.^[15]^ Indeed, ALF significantly increases the risk of cardiovascular complications and death in patients with MASLD, underscoring the urgent need for early identification and intervention in ALF. However, it is not clear whether hs-troponin can be used as a convenient and effective tool to predict ALF risk in individuals with MASLD without clinical CVD.

Systemic inflammation is recognized as a “common soil” for the development of various chronic diseases.^[16]^ It plays a pivotal role in the progression of MASLD and the formation of liver fibrosis.^[17]^ Evidence also suggests that hs-troponin levels are closely related to systemic inflammatory status.^[18,19]^ Therefore, inflammation might play a role in the association between hs-troponin levels and ALF in individuals with MASLD.

Accordingly, this study utilized data from the National Health and Nutrition Examination Survey (NHANES) among individuals aged 18 to 80 years, aiming to investigate the independent associations between hs-troponin (including T and I subtypes) and ALF in individuals with MASLD without CVD, to compare the predictive efficacy of different hs-troponin detection methods for ALF, and to evaluate the potential mediating role of systemic inflammatory status (represented by the monocyte-to-lymphocyte ratio [MLR]) in these associations.

## 2. Materials and methods

### 2.1. Study population

We utilized data from the 1999 to 2004 NHANES, a nationally representative survey conducted by the National Center for Health Statistics under the Centers for Disease Control and Prevention (CDC). NHANES employs a complex multistage probability sampling design to select noninstitutionalized American adults, enabling comprehensive assessment of health and nutritional status through household interviews, mobile examination center visits, and laboratory testing.

This study involved human participants. The survey protocols were reviewed and approved by the Institutional Review Board of the National Center for Health Statistics of the CDC (protocol number: protocol #98-12; NHANES 1999–2004).

**Figure 1.**
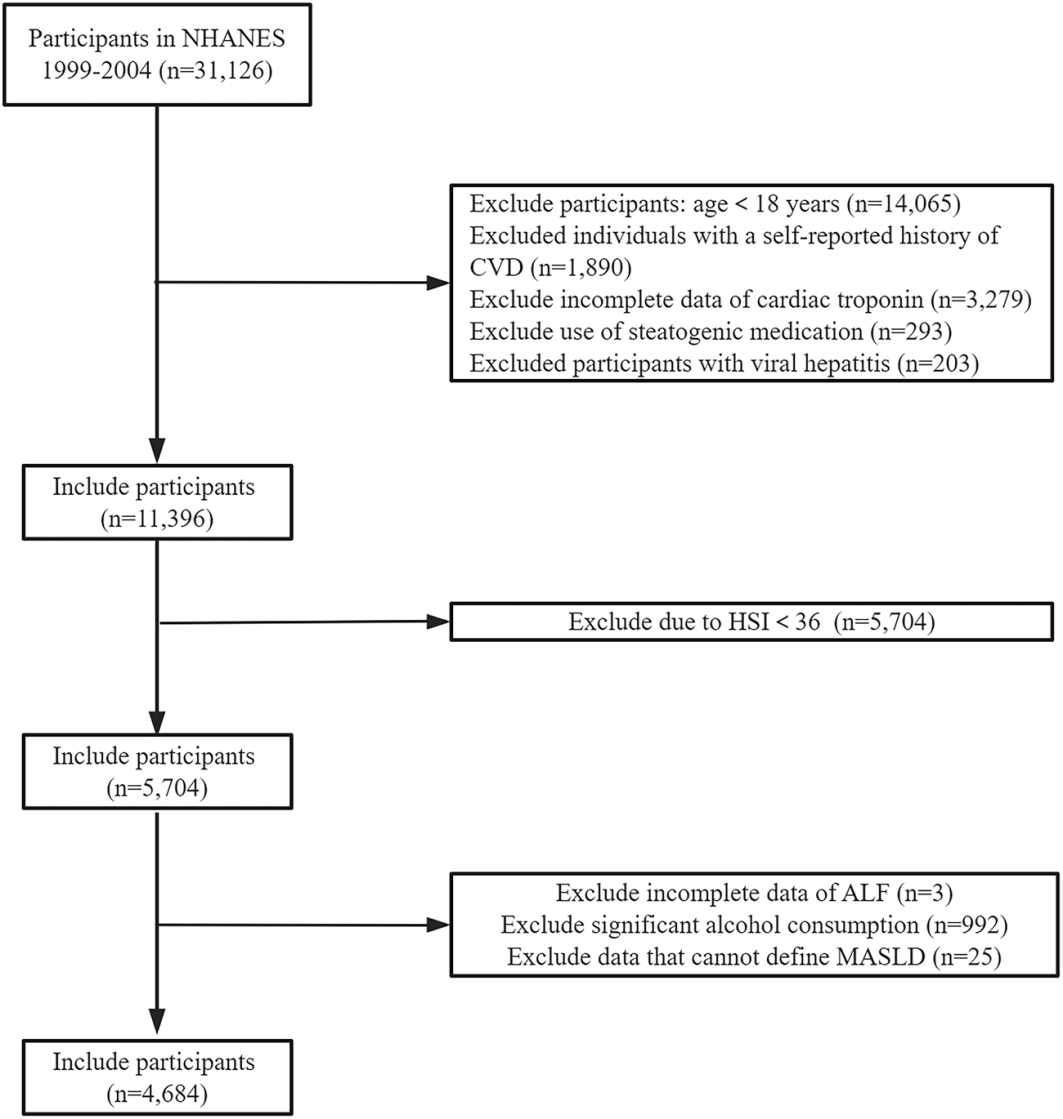
Flowchart of the study cohort selection from the National Health and Nutrition Examination Survey 1999 to 2004. ALF = advanced liver fibrosis, CVD = cardiovascular disease, HSI = Hepatic Steatosis Index, MASLD = metabolic dysfunction-associated steatotic liver disease.

From the initial 31,126 participants, we excluded individuals under 18 years of age, those with self-reported CVD (including congestive heart failure, coronary heart disease, angina, heart attack, or stroke), users of steatogenic medications (amiodarone, corticosteroids, methotrexate, tamoxifen, or valproate), heavy drinkers (defined as >2 drinks/day for women or >3 drinks/day for men in the past year), and participants with incomplete laboratory data (such as hs-troponin) or those lacking MASLD diagnostic information. The specific flowchart is shown in Figure 1.

### 2.2. Diagnosis of MASLD and ALF

Due to the absence of liver ultrasound or transient elastography data in NHANES 1999 to 2004, hepatic steatosis was assessed using the Hepatic Steatosis Index (HSI), calculated as follows^[20]^: HSI = 8 × (alanine aminotransferase [ALT]/aspartate aminotransferase [AST] ratio) + body mass index (BMI) + 2 (if female) + 2 (if diabetic). Individuals with HSI ≥36, without secondary causes of liver disease (excessive alcohol consumption, chronic liver disease etiologies, or steatogenic medications), were classified as having steatotic liver disease.^[20,21]^

MASLD is characterized by hepatic steatosis and requires the presence of 1 or more cardiometabolic criteria for diagnosis^[22]^: BMI≥25 kg/m^2^ (≥23 kg/m^2^ for Asian populations) or waist circumference (WC)>94 cm for men/>80 cm for women (adjusted for ethnicity); fasting plasma glucose ≥5.6 mmol/L, hemoglobin A1c (HbA1c) ≥5.7%, confirmed diagnosis of type 2 diabetes mellitus, or ongoing glucose-lowering therapy; blood pressure ≥130/85 mm Hg or use of antihypertensive medications; triglycerides ≥1.7 mmol/L or current lipid-lowering therapy; or high-density lipoprotein cholesterol (HDL-C) <1.0 mmol/L (men) or <1.3 mmol/L (women) or ongoing lipid-lowering therapy.

The diagnosis of ALF requires MASLD with a nonalcoholic fatty liver disease fibrosis score (NFS) ≥0.676 or a fibrosis-4 (FIB-4) score ≥2.67, or both. The NFS and FIB-4 are calculated as follows^[23]^: NFS = −1.675 + 0.037 × age (years) + 0.094 × BMI (kg/m^2^) + 1.13 × hyperglycemia (1 if yes, 0 if no) + 0.99 × AST/ALT − 0.013 × platelet count (×10^3^/mm^3^) − 0.66 × serum albumin (g/dL); FIB-4 = age (years) × AST (U/L)/(platelet count [×10^3^/mm^3^] × ALT [U/L])^1/2^. A higher NFS cutoff of 0.676 improves the precision of diagnosing advanced fibrosis, with positive predictive values of 90% in the derivation cohort and 82% in the validation cohort.^[24]^ Meanwhile, an FIB-4 score of ≥2.67 demonstrates an 80% positive predictive value for advanced fibrosis.^[25]^

### 2.3. Measurement of hs-troponin

From 1999 to 2004, the CDC stored serum samples from consenting NHANES participants for future research (approximately 88% of adults aged ≥18 years), maintaining them at −80°C until analysis. Between 2018 and 2020, the University of Maryland School of Medicine measured hs-troponin T (Roche) and hs-troponin I (Abbott, Siemens, and Ortho)^[26]^ in available samples from the NHANES 1999 to 2004 cohort. Consequently, hs-troponin testing was performed on all asymptomatic participants without clinical indications, with 93% of samples having no prior freeze-thaw cycles.^[26]^ hs-troponin T was measured using the Roche Cobas e601 with the fifth-generation Elecsys assay,^[26]^ while hs-troponin I was measured on ARCHITECT i2000SR (Abbott), Centaur XPT (Siemens), and Vitros 3600 (Ortho) platforms.^[26]^

### 2.4. Measurement of inflammatory markers

The Systemic Inflammation Index was derived from peripheral blood cell counts: MLR = monocytes/lymphocytes. This reflected systemic inflammatory status and correlated with increased cardiovascular risk.^[27]^ Fasting venous blood samples, collected after ≥9-hour overnight fasts, were analyzed using the Beckman Coulter DxH-800 for differential counts and the Cobas 6000 for HDL-C quantification. Standardized measurement protocols and bias assessments are documented in the NHANES Laboratory Procedure Manual.

### 2.5. Covariates

The covariates considered in this study included demographic information (age, gender, race/ethnicity, educational attainment, marital status, poverty-to-income ratio [PIR], and smoking status), physical and laboratory tests (BMI, WC, fasting blood glucose, HbA1c, ALT, AST, gamma-glutamyl transferase, total bilirubin, creatinine, total cholesterol, triglycerides, HDL-C, and low-density lipoprotein cholesterol), and comorbidities (history of diabetes, hypertension, and hyperlipidemia). Diabetes was defined as a self-reported prior diagnosis, HbA1c ≥6.5%, or fasting plasma glucose ≥7.0 mmol/L, and/or current use of insulin or oral hypoglycemic agents. Hypertension was defined as an average systolic blood pressure ≥140 mm Hg or diastolic blood pressure ≥90 mm Hg, or current use of antihypertensive drugs according to self-reported questionnaires. Other chronic comorbidities, such as hyperlipidemia, were determined based on a doctor’s diagnosis or self-reported questionnaires.

### 2.6. Statistical analysis

The NHANES data were collected using a complex, multistage probability design, including stratification, cluster sampling, and weighting adjustments. The purpose of these weights is to ensure that the sample accurately represents the noninstitutionalized US population. However, in this study, our focus was not on the representativeness of the participants but instead on the relationships between specific variables, such as hs-troponin and ALF. During the analysis, we chose not to apply the sampling weights, particularly considering that the data generation process had already been appropriately designed. Our primary interest lies in the internal structure of the models and hypothesis testing, rather than ensuring sample representativeness.

Continuous variables are presented as mean ± standard error and compared using *t* tests or one-way analysis of variance, while categorical variables were assessed with chi-square tests. To evaluate the relationship between hs-troponin T and 3 hs-troponin I with ALF in patients with MASLD, we constructed 3 multivariable logistic regression models: Model 1 was unadjusted for confounding factors; Model 2 was adjusted for age, sex, race/ethnicity, education level, PIR, and marital status; and Model 3 was further adjusted for smoking history, BMI, creatinine levels, diabetes, hypertension, and a history of hyperlipidemia based on Model 2. Based on the fully adjusted Model 3, subgroup analyses were conducted according to age, gender, race/ethnicity, smoking status, BMI, diabetes, hypertension, and hyperlipidemia, which verified the robustness of the results. The area under the receiver operating characteristic curve was estimated to determine the prognostic value of hs-troponin T for ALF in patients with MASLD, and the cutoff for hs-troponin T was estimated using the Youden Index. Additionally, mediation analysis was conducted using the “mediation” package in R, based on the fully adjusted logistic regression Model 3, to explore the mediating role of the inflammatory marker in the association between hs-troponin T and ALF. The average causal mediation effect, average direct effect, and the proportion of the mediation effect were calculated based on 1000 simulations. All statistical analyses were performed using R software (version 4.4.2; R Foundation for Statistical Computing), with a *P*-value < .05 considered statistically significant.

## 3. Results

### 3.1. Baseline characteristics of the study population

Figure 1 illustrates the screening process of the study population. The study included a total of 4684 patients with MASLD who had no history of CVD. The average age of the enrolled patients was 46.59 years, with 40.8% being male, and the prevalence of ALF was 3.6%. Table 1 presents the baseline characteristics of the study population. Patients in the ALF group were older, had lower levels of education, low to moderate household income, were former smokers, had higher BMI and WC, and had higher levels of blood glucose, AST, gamma-glutamyl transferase, and creatinine. They also had higher levels of hs-troponin and higher rates of diabetes, hypertension, and hyperlipidemia. No statistically significant differences were observed between the ALF and the non-ALF group in terms of sex, race/ethnicity, marital status, blood lipids, ALT, or total bilirubin levels.

**Table 1:** Baseline characteristics by ALF status in participants with MASLD.*

Characteristics	Overall (N = 4684)	Non-ALF (N = 4515)	ALF (N = 169)	*P*
Age (yr)	46.59 ± 18.38	45.69 ± 17.97	70.59 ± 11.98	<.001
Gender (%)				.261
Male	1911 (40.80)	1835 (40.64)	76 (44.97)	
Female	2773 (59.20)	2680 (59.36)	93 (55.03)	
Race/ethnicity (%)				.094
Mexican American	1227 (26.20)	1182 (26.18)	45 (26.63)	
Non-Hispanic White	955 (20.39)	921 (20.40)	34 (20.12)	
Non-Hispanic Black	2130 (45.47)	2045 (45.29)	85 (50.30)	
Other race	372 (7.94)	367 (8.13)	5 (2.96)	
Education (%)				<.001
<High school	678 (14.47)	627 (13.89)	51 (30.18)	
Completed high school	823 (17.57)	793 (17.56)	30 (17.75)	
>High school	3180 (67.89)	3092 (68.48)	88 (52.07)	
Not recorded	3 (0.06)	3 (0.07)	0 (0.00)	
Marital status (%)				.519
Married/living with partner	2826 (60.33)	2731 (60.49)	95 (56.21)	
Widowed/divorced/separated/never married	1697 (36.23)	1630 (36.10)	67 (39.64)	
Not recorded	161 (3.44)	154 (3.41)	7 (4.14)	
Poverty-to-income ratio (%)				<.001
<1.30	1267 (27.05)	1212 (26.84)	55 (32.54)	
1.30–3.5	1666 (35.57)	1592 (35.26)	74 (43.79)	
≥3.50	1396 (29.80)	1371 (30.37)	25 (14.79)	
Not recorded	355 (7.58)	340 (7.53)	15 (8.88)	
Smoking (%)				<.001
Never	2476 (57.81)	2393 (58.17)	83 (49.11)	
Current	527 (12.30)	521 (12.66)	6 (3.55)	
Former	1280 (29.89)	1200 (29.17)	80 (47.34)	
Body mass index (kg/m^2^)	32.43 ± 5.61	32.28 ± 5.43	36.53 ± 8.20	<.001
Waist circumference (cm)	105.85 ± 12.86	105.47 ± 12.58	116.37 ± 15.73	<.001
Diabetes mellitus (%)	749 (15.99)	628 (13.91)	121 (71.60)	<.001
Hypertension (%)	1817 (38.79)	1693 (37.50)	124 (73.37)	<.001
Hyperlipidemia (%)	1266 (27.03)	1203 (26.64)	63 (37.28)	.002
Lab panel				
Total cholesterol (mmol/L)	5.33 ± 1.11	5.33 ± 1.11	5.17 ± 0.97	.064
Triglycerides (mmol/L)	1.89 ± 1.48	1.89 ± 1.50	1.92 ± 1.16	.869
High-density lipoprotein cholesterol (mmol/L)	1.27 ± 0.36	1.27 ± 0.36	1.26 ± 0.37	.530
Low-density lipoprotein cholesterol (mmol/L)	3.19 ± 0.91	3.19 ± 0.91	3.01 ± 0.84	.087
Fasting glucose (mmol/L)	6.00 ± 2.18	5.93 ± 2.13	7.66 ± 2.66	<.001
Glycosylated hemoglobin (%)	5.70 ± 1.13	5.66 ± 1.11	6.62 ± 1.29	<.001
Alanine aminotransferase (U/L)	28.72 ± 36.33	28.77 ± 36.55	27.38 ± 29.92	.627
Aspartate aminotransferase (U/L)	24.51 ± 14.97	24.24 ± 13.68	31.59 ± 34.11	.006
Gamma-glutamyl transferase (U/L)	30.99 ± 39.56	30.72 ± 39.41	38.07 ± 42.82	.029
Albumin (g/L)	42.19 ± 3.76	42.24 ± 3.77	40.64 ± 3.14	<.001
Total bilirubin (μmol/L)	11.17 ± 4.49	11.16 ± 4.48	11.63 ± 4.50	.177
Creatinine (μmol/L)	71.47 ± 29.23	70.66 ± 26.34	93.06 ± 68.50	<.001
hs-troponin T, ng/L	7.04 ± 8.09	6.63 ± 7.05	17.91 ± 19.12	<.001
hs-troponin I (Abbott), ng/L	2.92 ± 6.26	2.79 ± 6.19	6.30 ± 7.14	<.001
hs-troponin I (Siemens), ng/L	5.44 ± 17.42	5.20 ± 17.06	11.93 ± 24.34	<.001
hs-troponin I (Ortho), ng/L	1.09 ± 4.33	1.01 ± 4.28	3.33 ± 5.17	<.001

ALF = advanced liver fibrosis, MASLD = metabolic dysfunction-associated steatotic liver disease.

* Values are mean ± SD or number of participants (percentage) unless otherwise indicated.

### 3.2. Relationship of the hs-troponin with ALF in patients with MASLD

Table 2 presents the results of multivariable logistic regression analysis of the association between hs-troponin T and 3 hs-troponin I and ALF in MASLD using 3 models. In Model 1, without adjustments, higher hs-troponin T and 3 hs-troponin I were significantly associated with increased odds of ALF. After adjusting for demographics (age, gender, race/ethnicity, educational attainment, PIR, and marital status) in Model 2, the association between hs-troponin T and ALF was attenuated but remained significant. Compared with those with lower hs-troponin T, patients with higher levels of hs-troponin T had an odds ratio of 1.03 (95% confidence interval [CI]: 1.01–1.04, *P* < .001) for ALF. In the fully adjusted Model 3, the association between hs-troponin T and ALF persisted. Individuals with higher levels of hs-troponin T had an odds ratio of 1.02 (95% CI: 1.01–1.03, *P* = .020) for ALF compared with those with lower hs-troponin T. However, the association between 3 hs-troponin I and ALF was not significant after adjusting for covariates. This suggests that hs-troponin T may have advantages over hs-troponin I in predicting ALF in the MASLD population, which requires further validation.

**Table 2:** Univariate and multivariable logistic models assessing the relationship between hs-troponin and ALF in patients with MASLD.

Variables	Model 1		Model 2		Model 3	
	OR (95% CI)	*P*	OR (95% CI)	*P*	OR (95% CI)	*P*
hs-troponin T	1.07 (1.06–1.09)	<.001	1.03 (1.01–1.04)	<.001	1.02 (1.01–1.03)	.020
hs-troponin I (Abbott)	1.03 (1.02–1.04)	<.001	1.00 (0.99–1.02)	.582	1.00 (0.98–1.02)	.848
hs-troponin I (Siemens)	1.01 (1.01–1.01)	<.001	1.00 (1.00–1.01)	.435	1.00 (0.99–1.01)	.647
hs-troponin I (Ortho)	1.04 (1.02–1.06)	<.001	1.00 (0.98–1.02)	.772	1.00 (0.97–1.02)	.803

Model 1: crude.

Model 2: adjusts for age, gender, race/ethnicity, educational attainment, poverty-to-income ratio, and marital status.

Model 3: adjusts for age, gender, race/ethnicity, educational attainment, poverty-to-income ratio, marital status, smoking status, body mass index, creatinine level, hypertension, hyperlipidemia, and diabetes mellitus.

ALF = advanced liver fibrosis, CI = confidence interval, MASLD = metabolic dysfunction-associated steatotic liver disease, OR = odds ratio.

### 3.3. Subgroup analysis

This study examined the association between hs-troponin T and ALF in patients with MASLD across subgroups defined by age, gender, race/ethnicity, BMI, smoking status, hypertension, hyperlipidemia, and diabetes mellitus, based on multivariate logistic regression Model 3 after adjusting for potential confounding factors (Table 3). No significant interaction was detected between hs-troponin T and the subgroups, indicating that the positive hs-troponin T-ALF relationship was consistent across diverse population segments. Overall, these findings demonstrate that higher levels of hs-troponin T are a robust independent risk factor for ALF irrespective of sociodemographic and lifestyle factors in US adults.

**Table 3:** Stratified analysis of the relationship between hs-troponin T and ALF in patients with MASLD.

Variables	OR (95% CI)	*P*	*P* for interaction
All patients	1.02 (1.00–1.03)	.020	
Age (yr)			.361
<60	1.01 (0.96–1.05)	.797	
≥60	1.02 (1.00–1.03)	.044	
Gender			.718
Female	1.02 (1.00–1.04)	.091	
Male	1.02 (1.00–1.03)	.065	
Race/ethnicity			.649
Mexican American	0.97 (0.91–1.04)	.416	
Non-Hispanic Black	1.02 (0.99–1.06)	.113	
Non-Hispanic White	1.02 (1.00–1.04)	.054	
Other	0.00 (0.00–Inf)	.993	
Smoking			.251
Never	1.01 (0.99–1.03)	.267	
Current	1.48 (1.03–2.14)	.035	
Former	1.02 (1.00–1.04)	.048	
BMI			.103
<30	1.01 (0.97–1.06)	.548	
≥30	1.02 (1.00–1.04)	.021	
Hypertension			.135
Yes	1.02 (1.01–1.04)	.007	
No	1.00 (0.98–1.02)	.995	
Hyperlipidemia			.262
Yes	1.01 (0.98–1.03)	.543	
No	1.02 (1.00–1.04)	.022	
Diabetes mellitus			.446
Yes	1.01 (0.99–1.03)	.359	
No	1.03 (1.00–1.05)	.079	

Stratified analysis was constructed based on logistic regression Model 3.

ALF = advanced liver fibrosis, BMI = body mass index, CI = confidence interval, MASLD = metabolic dysfunction-associated steatotic liver disease, OR = odds ratio.

### 3.4. hs-troponin T in predicting ALF in patients with MASLD

**Figure 2.**
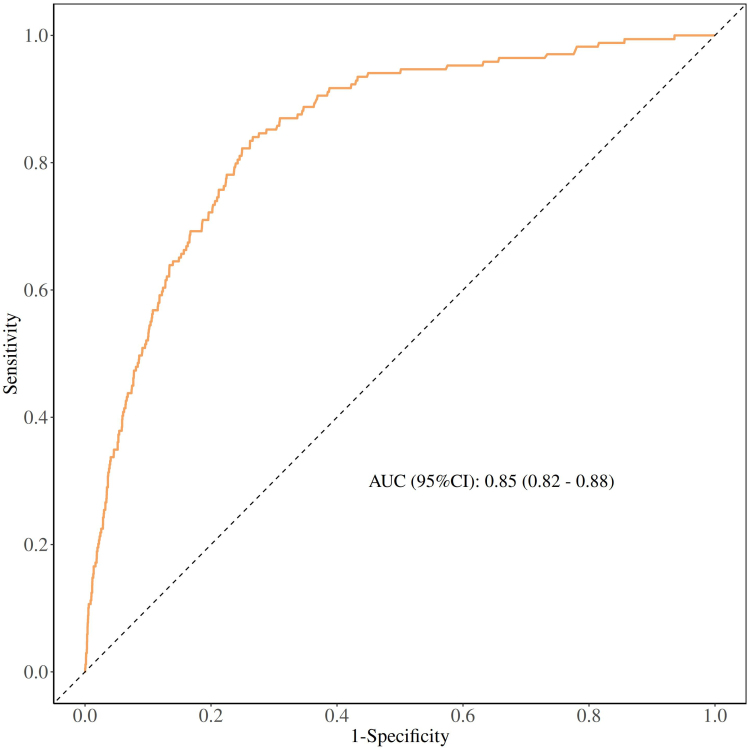
AUROC curve for hs-troponin T. AUC = area under the curve, CI = confidence interval.

In receiver operating characteristic analysis, the predictive value of the hs-troponin T index for ALF in patients with MASLD was illustrated in Figure 2 and Table 4. With the maximum Youden Index, the cutoff was set at 7.28 ng/L, with a sensitivity of 73% and specificity of 84% (area under the curve [AUC] = 0.85, 95% CI: 0.82–0.88, *P* *<* .001).

**Table 4:** Diagnostic performance of hs-troponin T for ALF in patients with MASLD.

	AUC	95% CI	Accuracy	Sensitivity	Specificity	Cutoff
hs-troponin T	0.85	0.82–0.88	74%	73%	84%	7.28

ALF = advanced liver fibrosis, AUC = area under the curve, CI = confidence interval, MASLD = metabolic dysfunction-associated steatotic liver disease.

### 3.5. Mediation analyses

**Figure 3.**
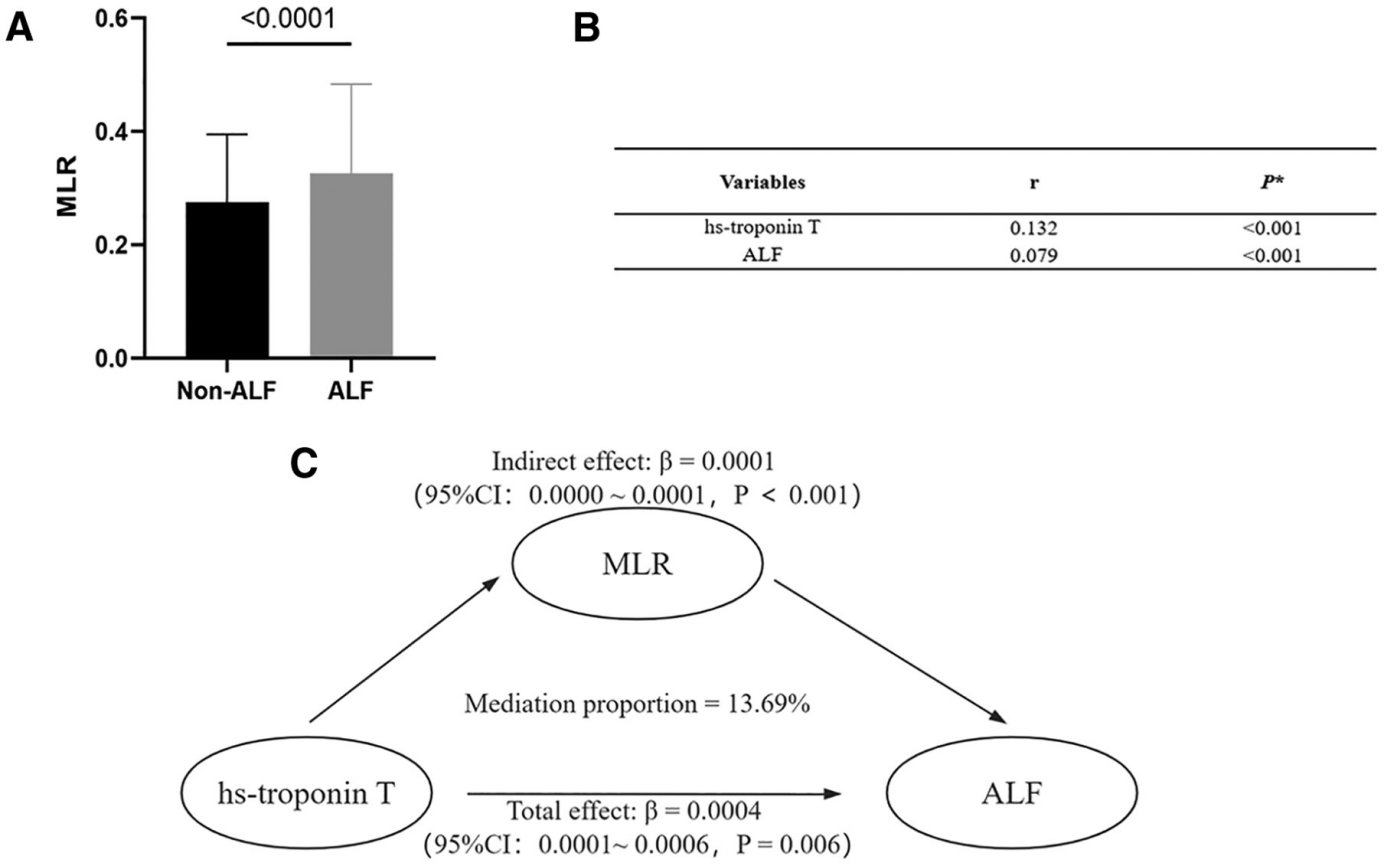
Role of monocyte-to-lymphocyte ratio (MLR) in the relationship between hs-troponin T and ALF in patients with MASLD. (A) Comparison of MLR between patients with and without ALF. (B) Correlations between MLR and hs-troponin T levels and ALF status. (C) The mediation effects of MLR on the association between hs-troponin T and ALF. ALF = advanced liver fibrosis, CI = confidence interval, MASLD = metabolic dysfunction-associated steatotic liver disease.

We observed that patients with MASLD and ALF had significantly higher MLR than those without ALF (*P* < .001; Fig. 3A). Moreover, MLR demonstrated positive correlations with both ALF status and hs-troponin T levels in the MASLD cohort (both *P* < .001; Fig. 3B). Furthermore, mediation analysis revealed that, after adjusting for confounding factors, MLR mediated the association between hs-troponin T and ALF, and the β-value of its mediating effect was 0.0001 (95% CI: 0.0000–0.0001; Fig. 3C). The mediating effect ratio was 13.69%, indicating that MLR mediated 13.69% of the association between hs-troponin T and ALF.

## 4. Discussion

This study, for the first time in a large cohort of patients with MASLD without clinical CVD, confirmed that plasma hs-troponin T levels were independently and positively associated with the risk of ALF, whereas no such significant association was observed with hs-troponin I. Second, we established a cutoff value of 7.28 ng/L for hs-troponin T in predicting ALF, which demonstrated good diagnostic performance (AUC = 0.85). Furthermore, the inflammatory marker MLR partially mediated (13.69%) the association between hs-troponin T and ALF. These findings suggest that hs-troponin T could emerge as a novel and practical tool for integrated risk stratification in MASLD.

hs-troponin T, primarily expressed in cardiomyocytes, exhibits elevated plasma levels during myocardial ischemia.^[28]^ However, nonischemic mechanisms – including intense inflammation and oxidative stress – can elevate hs-troponin T without acute cardiac events.^[29]^ Elevated levels are further documented in nonischemic conditions such as heart failure, left ventricular hypertrophy, chronic kidney disease, and diabetes.^[28]^ Given its robust association with cardiovascular events and mortality,^[7–9,29,30]^ hs-troponin T is increasingly recommended for CVD risk stratification. Population-based studies confirm its independent predictive value for CVD events and mortality, with similar prognostic significance established in CVD, chronic kidney disease, and diabetes cohorts.^[7–9,31]^

Our study identified an independent association between elevated hs-troponin T levels and ALF risk in patients with MASLD. Furthermore, we established a clinically relevant hs-troponin T cutoff value of 7.28 ng/L (AUC = 0.85) for predicting ALF in this population. These findings align with emerging hepatic evidence,^[10–12]^ which demonstrates that cirrhotic patients with Child-Pugh C classification exhibit significantly higher hs-troponin T levels than Class A patients, and that hs-troponin T serves as an independent predictor of long-term mortality, particularly in alcohol-related cirrhosis.^[12]^ Recent cohort studies extend this relationship to MASLD, confirming that hs-troponin T independently predicts all-cause and cardiovascular mortality regardless of baseline CVD status.^[10,11]^ Critically, MASLD and CVD exhibit bidirectional metabolic pathways^[32]^: progressive MASLD correlates with increased cardiometabolic risk and CVD severity, while advanced fibrosis predicts both hepatic and cardiovascular outcomes.^[33]^ As CVD remains the leading cause of death in patients with MASLD, independent of other comorbidities,^[5]^ hs-troponin T elevations – reflecting subclinical cardiovascular stress – may identify individuals concurrently at risk for hepatic fibrosis progression within this shared pathophysiological landscape. Our subgroup analyses further reinforce the robustness of the association between hs-troponin T and ALF.

Previous studies suggest that subclinical myocardial injury may contribute to liver fibrosis.^[34]^ Furthermore, the 2022 American Diabetes Association guidelines endorse biomarker-based approaches for subclinical disease risk assessment.^[35]^ Therefore, it is recommended that hs-troponin T be utilized for evaluating the risk of ALF in patients with MASLD. The hs-troponin T test is cost-effective, widely available, easy to use, and allows for prompt interpretation, enabling immediate cardiac risk stratification. This makes the hs-troponin T test a potentially feasible and viable addition to routine screening protocols for patients with MASLD. For patients with diagnosed MASLD, prioritizing assessment of disease activity and fibrosis stage remains essential^[15]^; however, incorporating hs-troponin T enables early identification of high-risk “asymptomatic” subgroups,^[36]^ cost-effective risk stratification preceding specialized liver assessments, and timely preventive interventions for both hepatic and cardiovascular complications. Enhanced accessibility to this test could significantly improve early detection and preventive care pathways. Prospective studies are warranted to validate the role of hs-troponin T in integrated hepatocardiac risk stratification algorithms.

MASLD is characterized by chronic low-grade inflammation, with significant oxidative stress accompanying its progression.^[37]^ Additionally, the development of ALF is closely associated with a systemic inflammatory state.^[17]^ Previous studies link hs-troponin T to inflammatory markers (such as C-reactive protein, inflammasomes, and interleukin-6).^[18,19]^ Our study further reveals that MASLD subjects with ALF exhibit significantly elevated MLR. Additionally, we observed significant positive correlations between MLR and both ALF status and hs-troponin T levels, aligned with previous studies. Meanwhile, basic research indicates shared pathological mechanisms between liver cirrhosis and cardiac dysfunction: systemic vasodilation abnormalities, renin-angiotensin-aldosterone system activation, and chronic inflammation constitute core pathways in hepatic-cardiac axis interactions.^[13]^ Within this framework, our mediation analysis demonstrates that MLR, as a systemic inflammation indicator, explains 13.69% of the positive association between hs-troponin T and ALF. This suggests that in individuals with MASLD but without CVD, the association between subclinical myocardial injury (reflected by elevated hs-troponin T) and ALF is partially mediated through inflammatory pathways.

Our study has several limitations. First, as this was a cross-sectional study, we were unable to establish a causal relationship between hs-troponin T and ALF. Second, the diagnosis of MASLD and ALF relied on noninvasive surrogates (HSI, NFS, and FIB-4) rather than gold-standard methods such as liver biopsy or vibration-controlled transient elastography. This might have led to misclassification and either an overestimation or an underestimation of the true associations. Future studies with more precise liver phenotyping are warranted to confirm our findings. Additionally, cardiovascular events (for exclusion) were based on self-reported data, which may introduce recall bias. Although we adjusted for a comprehensive set of covariates, unmeasured confounding factors (such as specific comorbidities) may still influence the observed associations. Finally, our findings may not be generalizable to other populations or racial/ethnic groups. Thus, multiracial and longitudinal studies and more sophisticated modeling approaches (e.g., causal inference methods) are needed to establish causality and further elucidate the role of hs-troponin T in ALF.

In summary, this study demonstrates that in a population with MASLD but without prevalent CVD, elevated hs-troponin T is associated with a higher risk of ALF. Furthermore, our findings suggest that inflammation may play a mediating role in the association between hs-troponin T and ALF. Therefore, elevated hs-troponin T in patients with MASLD may signal an increased risk of ALF, warranting closer monitoring. Additionally, screening for hs-troponin in individuals with MASLD but without active CVD may represent a more effective and beneficial approach to reducing adverse hepatic outcomes.

## Acknowledgments

The authors would like to express their gratitude for the valuable contributions made by the staff and participants of NHANES.

## Author contributions

**Conceptualization:** Chun Yu Li, Yun Xiu Wu, Kai Wang.

**Data curation:** Chun Yu Li, Yun Xiu Wu.

**Formal analysis:** Chun Yu Li, Yun Xiu Wu, Kai Wang.

**Methodology:** Chun Yu Li, Yun Xiu Wu, Kai Wang.

**Project administration:** Chun Yu Li.

**Software:** Chun Yu Li.

**Investigation:** Qing Zhang.

**Validation:** Qing Zhang.

**Visualization:** Qing Zhang.

**Resources:** Kai Wang.

**Supervision:** Kai Wang.

**Writing – original draft:** Chun Yu Li, Yun Xiu Wu.

**Writing – review & editing:** Chun Yu Li, Yun Xiu Wu, Kai Wang.
